# Determination of the protein quality of almonds (*Prunus dulcis* L.) as assessed by in vitro and in vivo methodologies

**DOI:** 10.1002/fsn3.1146

**Published:** 2019-07-29

**Authors:** James D. House, Kristen Hill, Jason Neufeld, Adam Franczyk, Matthew G. Nosworthy

**Affiliations:** ^1^ Department of Food and Human Nutritional Sciences University of Manitoba Winnipeg Manitoba Canada; ^2^ Department of Animal Science University of Manitoba Winnipeg Manitoba Canada; ^3^ Richardson Centre for Functional Food and Nutraceuticals University of Manitoba Winnipeg Manitoba Canada; ^4^ Canadian Centre for Agri‐Food Research in Health and Medicine, Albrechsten Research Centre St. Boniface General Hospital Winnipeg Manitoba Canada

**Keywords:** almond, in vitro protein digestibility, Protein Digestibility-Corrected Amino Acid Score, protein quality

## Abstract

Almonds (*Prunus dulcis*), such as all nuts, are positioned within the protein foods grouping within the current U.S. Dietary Guidelines. The ability to make claims related to the protein content of almonds, within the United States, requires substantiation via the use of the Protein Digestibility‐Corrected Amino Acid Score (PDCAAS). The present study was designed to provide current estimates of PDCAAS, using both in vivo and in vitro assays, of key almond varietals from the 2017 California harvest. Additionally, historical protein and amino acid composition data on 73 separate analyses, performed from 2000 to 2014, were analyzed. Amino acid analysis confirmed lysine as the first‐limiting amino acid, generating amino acid scores of 0.53, 0.52, 0.49, and 0.56 for Butte, Independence, Monterey, and Nonpareil varietals, respectively. True fecal protein digestibility coefficients ranged from 85.7% to 89.9% yielding PDCAAS values of 44.3–47.8, being highest for Nonpareil. Similar, albeit lower, results were obtained from the in vitro assessment protocol. Analysis of the historical data again positioned lysine as the limiting amino acid and yielded information on the natural variability present within the protein and amino acid profiles of almonds. Comparison of the 2017 AA profile, averaged across almond varietals, to the historical data provided strong evidence of persistence of amino acid composition and indices of protein quality over time.

## 1 INTRODUCTION

In the current United States dietary guidelines (U.S. Department of Health & Human Services, 2015), nuts fit within all of the currently recommended healthy dietary patterns, including the U.S.‐style Healthy Eating Pattern, the Healthy Mediterranean‐Style Eating Pattern, and the Healthy Vegetarian Eating Pattern. For each of these dietary patterns, nuts are positioned within the protein foods groups, due to their relative protein density. However, while nuts are grouped together within the same food group subcategory, their protein composition varies considerably. The USDA National Nutrient Database provides the following crude protein (% by weight) values for tree nuts: macadamia, 7.7%; pecans, 9.2%; Brazil nuts, 14.3%; walnuts, 15.2%; cashews, 18.2%; and pistachios, 20.2% (USDA, 2018). Almonds (*Prunus dulcis*) contain 21.2% protein by weight, with one 30 g serving of almonds providing 6.3 g of protein (Ahrens, Venkatachalam, Mistry, Lapsley, & Sathe, 2005; USDA, 2018). The latter represents approximately 16.6% and 13.4% of the Estimated Average Requirement for adult women and men, respectively (IOM, 2005).

While the current dietary guidelines recognize the importance of nuts, including almonds, to dietary protein intakes, communicating messages regarding the value of specific foods for the provision of protein necessitates meeting key regulatory requirements related to protein quality. In the United States, protein content claims on labels are based on the amount of quality‐corrected protein within a representative amount customarily consumed (RACC), where protein quality is assessed using the Protein Digestibility‐Corrected Amino Acid Score (PDCAAS) (US Food & Drug Administration, 2018). The latter is determined as the product of the Amino Acid Score (AAS) and the percent true fecal protein digestibility of the food in question. The AAS reflects the most limiting amino acid supplied by the food in comparison with an established reference requirement pattern and thus reflects a chemical assay. True protein digestibility, as stipulated by the FDA, requires the use of biological rodent assay to determine the amount of fecal nitrogen excreted per unit of dietary nitrogen consumed. The final PDCAAS values, which range from 0% to 100% (due to truncation) (FAO/WHO, 1991), are multiplied against the amount of crude protein in a RACC. In order to make protein content claims on foods, they must contain between 10% and 19% of the Daily Value (50 g) of PDCAAS‐corrected protein within a RACC for a “Good Source” claim, or 20% or greater for an “Excellent Source” claim. In the case of almonds (RACC = 30 g), the PDCAAS would need to be approximately 80% in order to qualify for a “Good Source of Protein” claim on labels. Attaining this value is a challenge for all nuts, including almonds, due primarily to limitations in their content of the indispensable AA lysine. As such, the requirement to attain the aforementioned quality benchmarks, as stipulated by the FDA, is somewhat incongruous to the message within the MyPlate dietary patterns regarding the importance of this food group in contributing protein to the human diet, a challenge described previously (Marinangeli & House, 2017).

Despite the above challenges, it is important to understand factors influencing the protein quality of almonds, as the sector seeks to position foods to satisfy the increasing demands by consumers for plant proteins. Published data by Ahrens et al. (2005), in 2005, provided an estimate for the AAS of almonds as being 0.26, with the sulfur AAs methionine and cysteine being limiting. Based on the AA content of almonds published in the USDA Nutrient Database, the AAS for almonds is calculated to be 0.46, with lysine being the limiting AA (USDA, 2018). Given these discrepancies, together with the interest in positioning plant‐based proteins to consumers, new estimates of the PDCAAS of almond varieties are warranted. The objectives of the current study were as follows: (a) to determine the PDCAAS (using both in vivo and in vitro estimates of protein digestibility) of 4 almond varietals and (b) to summarize historical protein and AA composition data on almonds gathered over 15 years, as generated by commercial laboratories.

## 2 MATERIALS AND METHODS

### 2.1 Statement on animal ethics

All procedures were approved by the Institutional Animal Care Committee (Protocol Number F2012‐035) in accordance with the guidelines of the Canadian Council on Animal Care (Canadian Council on Animal Care, 2018).

### 2.2 Materials

Composite samples (5,000 g) of shelled, raw Butte, Independence, Monterey, and Nonpareil almond varietals were provided by the Almond Board of California, Modesto, CA. All chemicals and reagents, including the NIST Standard Reference Material 3234 soy flour, were purchased from Sigma. Dietary ingredients for the in vivo true fecal protein digestibility study were procured from Dyets Inc..

### 2.3 Methods

#### 2.3.1 Sample preparation and analysis

Prior to the in vivo and in vitro analysis of the 4 almond varieties plus a casein (high nitrogen) control, all test articles were ground, using a Wiley Mill (Thomas Scientific) to pass through a 2‐mm screen. Subsamples for proximate and AA analysis were further ground, using a handheld electric mill, to pass through a 1‐mm screen. Samples were stored in airtight containers at −20**°**C prior to analysis.

The percent dry matter (AOAC Official Method 930.15), nitrogen (AOAC Official Method 968.06), crude fat (AOAC Official Method 2003.06; for diet formulation), ash (AOAC Official Method 950.49) of the test articles, and the final diets used in the in vivo PDCAAS assay (see below) were measured according to established procedures (Association of Official Analytical Chemists, 1995). For the measurement of most AA, with the exception of the sulfur AA methionine and cysteine, and tryptophan, samples were prepared as per AOAC Official Method 982.30, with 6N hydrochloric acid hydrolysis over 24 hr. Methionine and cysteine were analyzed according to AOAC Official Method 985.28, where proteins were first oxidized with performic acid prior to acid hydrolysis. For both hydrolysis sets, the AA was derivatized and separated (AccQ‐Tag Ultra C18, 1.7 µm column) using the AccQ‐Tag Ultra system (Waters Ltd., Mississauga, ON) chemistry (Astephen, 2018) on a Shimadzu UPLC system, complete with an SIL‐30AC autosampler. For tryptophan, samples were first subjected to alkaline hydrolysis and analyzed using ISO protocol 13904 (International Organization for Standardization, 2016). For quality control, the NIST soy flour Standard Reference Material 3234 was used for all AA analyses. For amino acid analyses, the hydrated molecular weights of amino acids were used for quantitation.

#### 2.3.2 Protein digestibility

In order to calculate PDCAAS for the test articles, the percent True Fecal Protein Digestibility (%TFPD) was measured according to AOAC Official Method 991.29 (Association of Official Analytical Chemists, 1995), with minor modifications to account for advances in rodent nutrition since the date of first action of the published method (1991). Principally, the AIN‐93G vitamin and mineral premixes were employed instead of the AIN‐76 formulations (Reeves, Nielsen, & Fahey, 1993), as the authors have described previously (House, Neufeld, & Leson, 2010; Nosworthy, Medina, et al., 2017). As an additional measure, the percent in vitro protein digestibility (%IVPD) was determined via the pH drop method, in duplicate, as previously described (Tinus, Damour, Riel, & Sopade, 2012).

#### 2.3.3 Protein quality calculations

The AAS was determined by comparing the AA composition of each test article with the recommended FAO/WHO reference pattern, reflecting the AA requirements of 2‐ to 5‐year‐old children (FAO/WHO, 2991) (mg/g protein: Histidine = 19; Isoleucine = 28; Leucine = 66; Lysine = 58; Threonine = 34; Tryptophan = 11; Valine = 35; Phenylalanine plus tyrosine = 63; Methionine plus cysteine = 25). Both the composition and reference patterns were first expressed in mg AA/g protein units. The lowest calculated AA ratio (limiting AA) was considered as the AAS. The final PDCAAS value was calculated as the product of the AAS and the %TFPD. In addition to the PDCAAS calculation, the in vitro PDCAAS was also determined as the product of the AAS and %IVPD (Nosworthy, Franczyk, et al., 2017).

#### 2.3.4 Historical analytical data procurement

Analytical data on the nutritional composition of 73 almond samples were provided by the Almond Board of California as final report printouts directly from commercial analytical laboratories (Covance; Medallion Labs). Analyses represented those performed between the years 2000 and 2014 (year/# of samples: 2000/5; 2001/1; 2002/8; 2003/5; 2004/1; 2005/23; 2006/1; 2008/5; 2009/17; 2014/7). The dataset represented a cross‐section of commercially available cultivars. Components analyzed varied by year and sample; however, data were available for all samples for dry matter, crude protein, and AA content. If crude protein was reported as *N* × 6.25, these were converted to values utilizing the established nitrogen conversion factor for almonds (*N* × 5.18; AOAC Official Method 968.06) (Association of Official Analytical Chemists, 1995). Tryptophan and the sulfur AA were not measured for all samples. The analytical laboratories are USDA‐certified and thus follow approved methodology for respective nutrient analyses. The AAS values were determined as described above. When tryptophan or the sulfur AA was absent, lysine was assumed to be the first‐limiting AA for AAS calculations.

#### 2.3.5 Statistical analyses

Data for composite samples are presented as the mean of duplicate analyses. Data for %TFPD were subjected to one‐way ANOVA, with *p*‐value <.05 taken to indicate significance and post hoc analyses conducted by Tukey's HSD method, using Prism 7 (GraphPad Software). Historical nutrient composition data were examined for outliers, using the nonlinear regression ROUT method (GraphPad Software), with a conservative false discovery rate of 0.1% and measures of central tendency and variation computed.

## 3 RESULTS AND DISCUSSION

### 3.1 Crude protein and amino acid composition of almonds

The nutritional profiles of the four composite almond varietal samples (Butte, Independence, Monterey, Nonpareil), from the 2017 California harvest year, are provided in Table 1. The mean (*SEM*) % content of protein and dry matter, across the four 2017 California harvest almond varieties, were 22.8 (0.9) and 4.3 (0.2), respectively. These data are consistent with the summary values published by USDA [2] (mean % [*SEM*]; protein = 21.2 [0.1]; moisture = 4.4 [0.2]), for almonds (USDA#12061). Ahrens et al. (2005) reported protein values for Carmel, Mission, and Nonpareil varieties of 20.6%, 23.3%, and 21.0%, with the latter value consistent with the Nonpareil protein value obtained in the current study (20.5%). In a study of the natural variability in California‐grown almonds (Yada, Huang, & Lapsley, 2013), seven varieties of almonds, including Butte, Monterey, and Nonpareil, were evaluated over 3 harvest years (2005–2007). In that study, harvest year, but not growing region, was found to have a significant impact on the protein content of the tested varieties (range 18.5%–24.0%). Evidence of the stability of varietal protein content is found in comparing the current data from the 2017 Nonpareil variety (20.5%) to data published in 1958, where protein content ranged between 19.9% and 20.3% (Hall, Moore, Gunning, & Cook, 1958). The latter research also provided evidence that processing, including blanching and roasting, did not impact protein content.

**Table 1:** Protein and amino acid composition of commercial almond varieties (2017 samples)

	Moisture	Crude protein^a^	Alanine	Arginine	Aspartate	Cysteine	Glutamate	Glycine	Histidine	Isoleucine	Leucine	Lysine	Methionine	Phenylalanine	Proline	Serine	Threonine	Tryptophan	Tyrosine	Valine
	%	%		%																
Butte	3.7	23.8	1.02	2.53	2.63	0.34	6.54	1.71	0.48	0.66	1.58	0.73	0.26	1.24	0.99	1.11	0.68	0.21	0.64	0.76
Independence	4.3	24.3	1.09	2.61	2.85	0.32	6.52	1.67	0.50	0.72	1.63	0.73	0.25	1.23	1.06	1.10	0.72	0.25	0.74	0.83
Monterey	4.8	22.7	1.00	2.45	2.70	0.28	6.12	1.60	0.47	0.68	1.48	0.65	0.22	1.15	0.95	0.99	0.66	0.21	0.67	0.78
Nonpareil	4.3	20.5	0.90	2.27	2.37	0.31	5.84	1.46	0.46	0.67	1.40	0.66	0.22	1.10	0.89	0.93	0.61	0.20	0.62	0.74
Casein	8.5	87.3	2.60	3.13	6.36	0.33	19.84	1.69	2.49	4.25	8.30	6.81	2.50	4.52	9.81	5.44	3.83	1.03	4.98	5.14
USDA—12061^b^	4.4	21.2	1.00	2.47	2.64	0.22	6.21	1.43	0.54	0.75	1.47	0.57	0.16	1.13	0.97	0.91	0.60	0.21	0.45	0.86

Note

Values are expressed as % by weight (as received basis).

a Crude protein for almond varieties was assessed as *N* × 5.18.

b Values derived from reference [2] for standard reference NDB# 12061, nuts, almonds.

While protein content is important, from a nutritional standpoint, the AA composition and the digestibility of food proteins are key to establishing the quality of the protein. The AA composition of the almond varietals is given in Table 1, expressed as a % as is (fresh weight) basis. The current data are in general agreement with those published by USDA [2], including the data for lysine and sulfur AA (methionine and cysteine). The latter is a critical point as it stands in stark contrast to the published values of Ahrens et al. (2005), who reported a sulfur AA content of 0.66 g/100 g protein, in contrast to the current study (average of 2.42 g/100 g protein). The explanation for the discrepancy likely lies in the fact that the previous authors did not appear to use the prerequisite performic acid oxidation step for the accurate measurement of the sulfur AA content (Association of Official Analytical Chemists, 1995) and thus greatly underestimated the total sulfur AA content.

### 3.2 Nutritional quality of almond protein

The determination of the AA profile is needed to establish the AAS (Table 2). For all almond samples, lysine was determined to be the limiting AA, resulting in an AAS ranging from 0.49 to 0.56. These data are consistent with the values calculated using the USDA Nutrient Database (0.463), but not with others (Ahrens et al., 2005), for reasons cited above. This AAS places almonds in a similar range (calculated from USDA, 2018) of AAS as walnuts (0.51) and pecans (0.54), along with other foods that are similarly limiting in lysine, including cereal grains and certain seeds, including hemp (House et al., 2010). Other plant proteins may have different limiting AAs, such as pulses, which tend to be limiting in either sulfur AA or tryptophan. For example, field peas present with an AAS of 0.8 (Nosworthy, Medina, et al., 2017). From a nutritional standpoint, blending almonds with complementary protein sources can enhance the final AAS of the meal/food product, and pulses may represent an opportune complementary protein class for almonds.

**Table 2:** Protein quality measures for commercial almond varieties (2017 samples)

	AAS^a^	%TFPD^b^	%IVPD^c^	PDCAAS^d^	IV‐PDCAAS^e^	Wt. gain/protein intake^f^	Adj. wt. gain/protein intake^g^
Butte	0.530	86.2 (1.1)^cd^	78.3	45.7	41.5	1.56 (0.11)^bc^	1.42
Independence	0.519	88.9 (0.6)^bc^	78.9	46.2	40.9	1.44 (0.06)^c^	1.31
Monterey	0.493	89.9 (0.3)^b^	80.6	44.3	39.7	1.17 (0.10)^c^	1.06
Nonpareil	0.557	85.7 (0.7)^d^	78.6	47.8	43.8	1.92 (0.10)^b^	1.75
Casein	1.075	96.0 (0.7)^a^	89.0	100.0	95.7	2.75 (0.12)^a^	2.50

Note

Values are means (*SEM*). Values within a column with different superscripts are significantly different by Tukey's multiple comparisons test.

a AAS = amino acid score, based on the reference AA requirement pattern for 2‐ to 5‐year‐old children.

b %TFPD = true fecal protein digestibility.

c %IVPD = in vitro protein digestibility.

d PDCAAS = Protein Digestibility‐Corrected Amino Acid Score. Values > 100 are truncated to 100.

e IV‐PDCAAS = in vitro Protein Digestibility‐Corrected Amino Acid Score.

f Body weight gain (g)/protein intake (g) over 9‐day digestibility study period (NB: not an official estimate of PER).

g Body weight gain (g)/protein intake (g) over 9‐day digestibility study, corrected for casein and standardized to 2.5.

The AAS of almonds represents one component of protein quality. The digestibility of the protein and ultimate utilization of the constituent AAs for metabolic functions is equally important in establishing quality. Data on the estimates of %TFPD and %IVPD are presented in Table 2. Of the four almond varietals tested, %TFPD was highest for Monterey and lowest for Butte, with the difference ranging by 4 percentage units. While the absolute values differ, the pattern of response for the %IVPD was similar to that of the %TFPD. These values led to PDCAAS and IV‐PDCAAS values ranging between 44.3–47.8 and 39.7–43.8, respectively (lowest for Monterey; highest for Nonpareil). As an additional measure of protein quality, body weight gain (g) per g of protein consumed was calculated. This value is consistent with the protein efficiency ratio (PER); however, the measurement period of 9 days (fecal collection period) is technically too short for a PER protocol (Association of Official Analytical Chemists, 1995). Despite a limited dataset, the agreement between the PDCAAS values and the body weight gain per unit protein consumed was observed with *r* ^2^ of .92. These results highlight the ability of almonds to contribute to the growth of young rodents, with differences between almond varietals explained, at least in part, by differences in the AA profile and factors influencing protein digestibility. Previous research has documented that the PER of Nonpareil almonds, which was 1.62 for blanched almonds, was decreased to 1.12, 1.02, and 0.24 as a result of dry‐roasting, oil‐roasting, and toasting, respectively (Hall et al., 1958). Further efforts should be placed to understand the impact of almond processing on measures of protein and AA digestibility. The latter may be more important as efforts have been made to position a new method for assessing protein quality, based on the Digestible Indispensable Amino Acid Score (DIAAS) system (FAO/WHO, 2013). This method requires a more invasive approach to measuring ileal AA digestibility and may prove challenging to implement for a diverse and complex human foodscape. Given the potential for in vitro approaches to approximate in vivo digestibility coefficients, efforts should be placed on validating and approving in vitro approaches for assessing protein quality.

In an effort to further explore variation in the protein and AA content of almonds, data from 73 individual sample analysis submissions made to commercial laboratories were evaluated (Table 3). The data provide an overview of the ranges of protein and amino acids reported for almonds over the last two decades, for a mix of almond varieties. Plotting the average of the 4 values for the 2017 samples for each AA against the corresponding mean value for AA from Table 3 yielded a slope estimate of 1.11 (*SEM* = 0.02), an intercept of −81.29 (*SEM* = 38.15), and *r* ^2^ value of .99. The latter result reflects general agreement between the analytical methods used in the current study to those used in commercial laboratories and also confirms the resiliency of the protein and AA content of the major almond varieties over time.

**Table 3:** Proximate analysis, crude protein (nitrogen × 5.18), and amino acid content of almond samples analyzed between the years 2000 and 2014 in commercial laboratories

	*N* a	Minimum	Median	Maximum	Mean	Standard error	Coefficient of variation
Moisture (%)	73	1.97	4.50	6.30	4.36	0.095	18.6
Fat (%)	73	39.5	49.6	57.0	49.37	0.305	5.3
Fiber (%)	72	7.90	12.40	19.30	12.20	0.238	16.6
Carbohydrates (%)^b^	58	16.80	23.50	34.50	23.69	0.448	14.4
Ash (%)	72	2.17	2.90	3.70	2.90	0.026	7.8
Protein (%)	70	16.82	20.50	23.95	20.56	0.132	5.4
Alanine (%)	70	0.48	1.01	1.98	1.10	0.040	30.3
Arginine (%)	72	1.18	2.37	3.27	2.36	0.042	15.2
Aspartate (%)	73	1.27	2.39	3.90	2.50	0.061	20.9
Cysteine (%)	49	0.18	0.27	0.34	0.26	0.005	13.9
Glutamate (%)	73	3.01	5.67	7.77	5.60	0.101	15.4
Glycine (%)	73	0.91	1.48	2.16	1.48	0.029	17.0
Histidine (%)	73	0.27	0.51	0.63	0.50	0.009	15.4
Isoleucine (%)	71	0.54	0.83	0.99	0.82	0.011	11.1
Leucine (%)	67	1.20	1.51	1.87	1.52	0.014	7.6
Lysine (%)	73	0.35	0.62	0.71	0.59	0.010	14.2
Methionine (%)	71	0.07	0.19	0.23	0.18	0.004	18.0
Phenylalanine (%)	69	0.83	1.17	1.39	1.16	0.013	9.6
Proline (%)	73	0.56	0.90	1.13	0.88	0.016	15.2
Serine (%)	71	0.55	0.89	1.33	0.88	0.015	14.7
Tryptophan (%)	28	0.09	0.18	0.23	0.18	0.006	18.5
Tyrosine (%)	72	0.36	0.65	0.85	0.65	0.010	13.0
Valine (%)	72	0.59	0.94	1.27	0.93	0.016	14.1
Amino acid score	73	0.29	0.51	0.71	0.51	0.009	15.2

Note

Data presented on “as is” basis.

Differences in *N* from the maximum (73) represent either missing values or values removed via outlier analysis. Number of outliers removed: Ash = 1; Dietary Fiber = 1; Protein = 3; Threonine = 8; Serine = 2; Alanine = 3; Valine = 1; Isoleucine = 2; Leucine = 6; Tyrosine = 1; Phenylalanine = 4; Cysteine = 1; Methionine = 2. ^2^Lysine = limiting amino acid.

a *N* = sample size.

b Carbohydrates determined by difference.

## 4 CONCLUSION

In conclusion, the current data support a PDCAAS value for raw almonds of between 44.3 and 47.8, for the varieties tested. Concurrent assessment of in vitro digestibility and growth provided additional evidence of the quality of almond proteins. The data can be used to guide varietal selection for amino acid content; however, the natural variability between varieties, particularly in lysine content, may not be sufficient to make substantial improvements. Of the varieties test, Nonpareil presented with the most consistent pattern of highest protein quality. Given the importance of Nonpareil to the California almond industry, the current results support continued attention to this variety.

## CONFLICT OF INTEREST

The authors declare no conflicts of interest with regard to the described research, the publication of results, or financial issues.

## AUTHOR CONTRIBUTION

JDH designed and oversaw the experimental components of the study, and prepared the first draft of the manuscript. KH, JN, AF, and MGN conducted the technical analyses of the samples and conducted the animal experimentation for the in vivo protein digestibility coefficients. KH entered the historical compositional data, and JDH verified its integrity.

## ETHICAL APPROVAL

All procedures involving animals were approved by the Institutional Animal Care Committee (Protocol Number F2012‐035) in accordance with the guidelines of the Canadian Council on Animal Care (Canadian Council on Animal Care, 2018).

## References

[fsn31146-bib-0001] Ahrens, S. , Venkatachalam, M. , Mistry, A. M. , Lapsley, K. , & Sathe, S. K. (2005). Almond (*Prunus dulcis* L.) protein quality. Plant Foods for Human Nutrition, 60, 123–128. 10.1007/s11130-005-6840-2 16187015

[fsn31146-bib-0002] AOAC Official Methods of Analysis (1995). Association of Official Analytical Chemists. Arlington, Washington, DC: AOAC.

[fsn31146-bib-0003] Astephen, N. (2018). Waters AccQ‐Tag method for hydolysate amino acid analysis. Retrieved from http://www.waters.com/waters/library.htm?cxml:id=511436&lxml:id=1520760&locale=120

[fsn31146-bib-0004] Canadian Council on Animal Care (2018). Guidelines for the care of laboratory rats. Retrieved from https://www.ccac.ca/en/standards/guidelines/types-of-animals.html

[fsn31146-bib-0005] FAO/WHO (1991). Protein quality evaluation. Report of the joint FAO/WHO expert consultation. Food and nutrition paper no. 51. Rome, Italy: Food and Agriculture Organizations and the World Health Organization.

[fsn31146-bib-0006] FAO/WHO (2013). Dietary protein quality evaluation in human nutrition: Report of an FAO expert consultation. Food and nutrition paper no. 92. Rome, Italy: Food and Agriculture Organizations and the World Health Organization. 26369006

[fsn31146-bib-0007] Hall, A. P. , Moore, J. G. , Gunning, B. , & Cook, B. (1958). The nutritive value of fresh and roasted California‐grown Nonpareil almonds. Journal of Agricultural and Food Chemistry, 6, 377–382. 10.1021/jf60087a008

[fsn31146-bib-0008] House, J. D. , Neufeld, J. , & Leson, G. (2010). Evaluating the quality of protein from hemp seed (*Cannabis sativa* L.) products through the use of the Protein Digestibility-Corrected Amino Acid Score. Journal of Agricultural and Food Chemistry, 58, 11801–11807. 10.1021/jf102636b 20977230

[fsn31146-bib-0009] Institute of Medicine (2005). Dietary reference intakes for energy, carbohydrates, fiber, fat, fatty acids, cholesterol, protein, and amino acids. Washington, DC: National Academies Press.

[fsn31146-bib-0010] International Organization for Standardization (2016). Animal feeding stuffs – Determination of tryptophan content ISO 13904. Geneva, Switzerland: International Organization for Standardization.

[fsn31146-bib-0011] Marinangeli, C. P. F. , & House, J. D. (2017). Potential impact of the digestible indispensable amino acid score as a measure of protein quality on dietary regulations and health. Nutrition Reviews, 75, 658–667. 10.1093/nutrit/nux025 28969364 PMC5914309

[fsn31146-bib-0012] Nosworthy, M. G. , Franczyk, A. , Zimoch‐Korzycka, A. , Appah, P. , Utioh, A. , Neufeld, J. , & House, J. D. (2017). Impact of processing on the protein quality of pinto bean (*Phaseolus vulgaris*) and buckwheat (*Fagopyrum esculentum Moench*) flours and blends, as determined by *in vitro* and *in vivo* methodologies. Journal of Agricultural and Food Chemistry, 65, 3919–3925. 10.1021/acs.jafc.7b00697 28452476

[fsn31146-bib-0013] Nosworthy, M. G. , Medina, G. , Franczyk, A. J. , Neufeld, J. , Appah, P. , Utioh, A. , … House, J. D. (2017). Effect of processing on the in vitro and in vivo protein quality of yellow and green split peas (*Pisum sativum*). Journal of Agricultural and Food Chemistry, 65, 7790–7796. 10.1021/acs.jafc.7b03597 28796503

[fsn31146-bib-0014] Reeves, P. G. , Nielsen, F. H. , & Fahey, G. C. Jr (1993). AIN‐93 purified diets for laboratory rodents: Final report of the American Institute of Nutrition ad hoc writing committee on the reformulation of the AIN‐76A rodent diet. Journal of Nutrition, 123, 1939–1951. 10.1093/jn/123.11.1939 8229312

[fsn31146-bib-0015] Tinus, T. , Damour, M. , Van Riel, V. , & Sopade, P. A. (2012). Particle size‐starch‐protein digestibility relationships in cowpea (*Vigna unguiculata*). Journal of Food Engineering, 113, 254–264. 10.1016/j.jfoodeng.2012.05.041

[fsn31146-bib-0016] U.S. Department of Health and Human Services and U.S. Department of Agriculture (2015). 2015–2020 Dietary guidelines for Americans (8th ed.). Washington, DC: U.S. Department of Health and Human Services and U.S. Department of Agriculture.

[fsn31146-bib-0017] US Food and Drug Administration (2018). Code of federal regulations Title 21. Retrieved from https://www.accessdata.fda.gov/scripts/cdrh/cfdocs/cfcfr/CFRSearch.cfm?fr=101.54

[fsn31146-bib-0018] USDA National Nutrient Database for Standard Reference, Legacy Release (2018). Retrieved from https://ndb.nal.usda.gov/ndb/search/list?home=true

[fsn31146-bib-0019] Yada, S. , Huang, G. , & Lapsley, K. (2013). Natural variability in the nutrient composition of California‐grown almonds. Journal of Food Composition and Analysis, 30, 80–85. 10.1016/j.jfca.2013.01.008

